# Advances in multimodal treatment for stage IIIA-N2 non-small cell lung cancer

**Published:** 2021-04-16

**Authors:** Sara Montemuiño Muñiz, Soraya Marcos Sánchez, Julia Calzas Rodríguez, Beatriz Losada Vila, Esther Llorente Herrero, María Dolores Hisado Díaz, Victoria Valeri-Busto González, Begoña Taboada Valladares, Blanca Vaquero Barrón, Francisco José Marcos Jimenez, Sergio Amor Alonso, Javier Moradiellos, Núria Rodríguez de Dios, Felipe Couñago

**Affiliations:** ^1^Department of Radiation Oncology, Hospital Universitario de Fuenlabrada, Camino del Molino, 2, 28942, Fuenlabrada, Madrid, Sara Montemuiño, Spain; ^2^Department of Medical Oncology, Hospital Universitario de Fuenlabrada, Camino del Molino, 2, 28942, Fuenlabrada, Madrid, Spain; ^3^Department of Nuclear Medicine, Hospital Universitario de Fuenlabrada, Camino del Molino, 2, 28942, Fuenlabrada, Madrid, Spain; ^4^Department of Pulmonology, Hospital Universitario de Fuenlabrada, Camino del Molino, 2, 28942, Fuenlabrada, Madrid, Spain; ^5^Department of Radiation Oncology, Complexo Hospitalario Universitario Santiago de Compostela, Choupana s/n, bloque d, Santiago de Compostela, A Coruña, Spain; ^6^Department of Radiation Oncology, Hospital Universitario de La Princesa, C/Diego de León, 62, 28006, Madrid, Spain; ^7^Department of Radiation Oncology, Hospital Universitario de Cáceres, Avda, Universidad 75, 10004, Cáceres, Extremadura, Spain; ^8^Department of Thoracic Surgery, Hospital Universitario Quirónsalud Madrid, C/Diego de Velázquez, 1, 28223, Pozuelo de Alarcón, Madrid, Spain; ^9^Department of Radiation Oncology, Hospital del Mar, Passeig Marítim, 25-29, 08003 Barcelona, Spain; ^10^IMIM (Hospital del Mar Medical Research Institute), Barcelona, Spain; ^11^Pompeu Fabra University, Barcelona, Doctor Aiguader, 80, 08003 Barcelona; ^12^Department of Radiation Oncology, Hospital Universitario Quirónsalud Madrid, C/Diego de Velázquez, 1, 28223, Pozuelo de Alarcón, Madrid, Spain; ^13^Hospital La Luz, Calle del Maestro Ángel Llorca 8, 28003, Madrid, Spain; ^14^Universidad Europea de Madrid, Calle Tajo, s/n, 28670 Villaviciosa de Odón, Madrid, Spain

**Keywords:** chemoradiation, immunotherapy, neoadjuvant treatment, non-small cell lung cancer, stage IIIA, surgery

## Abstract

**Background and Aim:**

In Stage IIIA-N2 non-small cell lung cancer (NSCLC), the accuracy of combined positron-emission tomography/computed tomography imaging (PET-CT), together with mediastinal staging techniques, has led to a wide range of challenging clinical scenarios in terms of therapeutic management. Concurrent chemoradiotherapy followed by consolidation immunotherapy remains the standard of care. In patients with potentially-resectable disease, surgery plays an important role in multimodal therapy. The introduction of targeted therapies and immune-checkpoint inhibitors has revolutionized multimodal treatment. In the present article, we review current treatment options and future trends in stage IIIA-N2 NSCLC.

**Relevance for Patients:**

This article provides insight into the current status of multimodal treatment for NSCLC to support decision-making in routine clinical practice.

## 1. Introduction

Lung cancer is the leading cause of cancer related-death worldwide in both sexes [1]. Non-small cell lung cancer (NSCLC) accounts for approximately 85% of all lung cancer diagnoses [2]. Imaging tests are used to characterize the primary lesion and to detect mediastinal involvement and distant disease. However, due to late symptom onset, more than one-third of patients present locally-advanced disease at diagnosis.

Stage IIIA-N2 NSCLC comprises a highly heterogeneous group of patients, with poor 5-year survival rates, approximately 37% in patients with cN1 disease and 23% in those with cN2 disease [3]. The accuracy of mediastinal staging is crucial in patients with suspected NSCLC, not only for its prognostic value but also to select the most appropriate multimodal treatment. Cytological/histological confirmation of mediastinal involvement is essential [4].

The therapeutic management of Stage IIIA-N2 disease is challenging due to the wide range of clinical scenarios. Concurrent chemoradiotherapy (CRT) followed by consolidation immunotherapy remains the standard treatment for patients with inoperable or unresectable disease [5,6]. However, in a subset of patients with potentially-resectable Stage IIIA-N2 disease, the addition of surgery to the multimodal treatment approach appears to improve local control and survival, provided that extensive resections are avoided [7].

The optimal treatment for patients with Stage IIIA-N2 NSCLC is a subject of intense debate among thoracic surgeons, radiation oncologists, and medical oncologists. Moreover, the emergence of targeted therapies and immunotherapy has transformed treatment decision-making. The objective of the present review is to provide an overview of current treatment options.

## 2. Diagnostic aspects of positron-emission tomography-computed tomography (PET-CT)

CT has long been the diagnostic method of choice in lung cancer due to its widespread availability, its ability to identify the morphological characteristics of the primary tumor and the involvement of adjacent structures, and because it can detect mediastinal involvement as well as distant disease. The emergence of 18F-fluorodeoxyglucose (FDG) PET combined with CT (PET-CT) has changed the paradigm of oncological imaging in the diagnosis of lung cancer. This imaging modality provides highly accurate anatomical and metabolic data in a single imaging study. As a result, PET-CT in now a standard tool for the diagnosis and staging of lung cancer, as well as for re-staging patients with recurrent disease. Moreover, PET-CT is also used to guide treatment, assess treatment response, and for prognostic purposes [8,9].

Compared to conventional imaging techniques such as CT, 18F-FDG PET/CT offers important advantages in staging mediastinal and extrathoracic disease, with a 51% relative reduction in unnecessary thoracotomies compared to conventional methods; in other words, the use of 18F-FDG PET/CT can obviate the need for one out of every five surgeries [10]. For the diagnosis of mediastinal lymph node disease, the sensitivity and specificity of 18F-FDG PET-CT are approximately 62–72% and 89–94%, respectively [11-13]. Darling *et al*. conducted a randomized controlled trial (RCT) to evaluate the diagnostic efficacy of 18F-FDG PET/CT for mediastinal staging, finding a clinically-relevant false positive rate of around 35%, mainly due to the presence of granulomatous and/or other inflammatory phenomena. This limitation confirms the need for pathological confirmation of mediastinal lymph node abnormalities detected on 18F-FDG PET/CT [11,14].

The molecular information obtained with 18F-FDG PET/CT allows us to discriminate atelectasis from tumor-related obstructive pneumonitis, allowing for more accurate delineation of the radiotherapy target volume [15,16]. A systematic review found that the target definition was significantly altered in approximately two out of every five patients (40%), a finding that underscores the need to perform PET-CT before radiation treatment planning [17].

PET/CT also provides valuable functional information to assess treatment response. This imaging technique can detect metabolic changes earlier than morphological alterations, thus improving diagnostic accuracy and permitting early diagnosis. A meta-analysis found that 18F-FDG PET/CT has a significantly higher predictive value than CT for pathological response after neoadjuvant treatment in patients with NSCLC. Furthermore, the high negative predictive value (NPV) of 18F-FDG PET/CT (91%) can help to identify patients whose disease does not respond to treatment [18].

The use of 18F-FDG PET/CT for follow-up is not standard, but given the importance of detecting recurrences as early as possible for optimal treatment, this imaging modality could improve both survival outcomes as well as quality of life (QoL). A meta-analysis carried out by He *et al*. to compare the diagnostic efficacy of PET, PET/CT, and conventional imaging found that although PET and PET/CT had good sensitivity and specificity (around 90%), hybrid PET/CT imaging offered greater diagnostic accuracy [19].

## 3. Invasive mediastinal staging

Cytological/histological confirmation of mediastinal involvement in patients with abnormal imaging tests and/or in those with a high risk of mediastinal involvement can be performed with surgical techniques such as mediastinoscopy, which was the gold standard until a few years ago, or through minimally-invasive endosonographic techniques such as real-time endobronchial ultrasound-guided transbronchial needle aspiration (EBUS-TBNA) or esophageal endoscopic ultrasound with fine-needle aspiration (EUS-FNA).

### 3.1. Minimally-invasive techniques

Endosonography (EBUS-TBNA and/or EUS-FNA) has been proposed as the initial imaging modality for mediastinal staging and diagnostic confirmation instead of surgical techniques [4,20]. The sensitivity of endoscopic ultrasound for the detection of mediastinal metastases (followed by surgery, if negative) is 94% versus 79% for surgical staging alone, with a NPV of 93% versus. 86%, respectively [21]. The use of these endosonographic techniques, either alone or in combination, is equivalent to surgical techniques, with a sensitivity, NPV, and diagnostic accuracy of approximately 90%, a high specificity (nearly 100%), and a low complication rate [22-27].

The combined use of EBUS-TBNA and EUS-FNA has been shown to provide greater diagnostic efficacy and accuracy than either technique alone, with a significant improvement in sensitivity and NPV for the detection of metastases [28,29]. For this reason, clinical guidelines recommend the combined use of these techniques whenever possible, together with the systematic sampling of at least three mediastinal stations (paratracheal, subcarinal, and hilar) [4] since this combined approach improves the diagnostic sensitivity by 4% (EBUS) and 9% (EBUS + EUS) versus PET-CT alone in cases of suspected nodal involvement [28,30,31]. Moreover, these techniques are safe, with very low morbidity and mortality rates, cost-effective, accurate, and rapid – providing diagnostic information in less time with fewer invasive tests [32]. However, in cases with negative results on endoscopic imaging but in which there is a strong suspicion of nodal involvement, surgical staging is usually required [25,33].

Mediastinal re-staging after induction therapy is controversial and difficult due to the presence of fibrosis, adhesions, and tissue necrosis. The latest meta-analyses show that the diagnostic precision of endosonographic imaging for restaging is lower than for the initial staging, with a sensitivity ranging from 63–77%, although specificity remains high (99%) [34,35].

### 3.2. Surgical techniques

Due to advances in imaging and endosonographic imaging, surgical techniques are, in most cases, no longer considered the first step in the diagnostic algorithm. However, surgery continues to play an important role in mediastinal staging of potentially-resectable NSCLC as a confirmatory technique in cases in which the endosonography is negative for mediastinal involvement but there is a high suspicion of nodal involvement; surgical techniques are also useful to reach stations that cannot be accessed with minimally-invasive techniques, such as nodal stations 5 and 6 in left lung tumors, and for re-staging after induction therapy [36,37]. Both the American College of Chest Physicians and the European Society of Thoracic Surgeons guidelines recommend exploring at least five mediastinal nodal stations (2R, 2L, 4R, 4L, and 7) and biopsying at least one node from each station, as well biopsying stations five and five in left lung cancer [25,38].

Video-assisted mediastinoscopy (VAM) has steadily gained ground over conventional mediastinoscopy due to better visualization of the lymph node stations and the potential for more extensive mediastinal sampling (reaching station 7). VAM shows good sensitivity and NPV (89% and 96%, respectively), which is slightly better than those obtained with conventional mediastinoscopy (83% and 90%, respectively) [25]. Parasternal mediastinotomy and extended cervical mediastinoscopy have shown same sensitivity and NPV, 71% and 91%, respectively, for the exploration of nodal stations 5 and 6 [39]. A more invasive alternative to these two techniques is video-assisted thoracoscopic surgery (VATS), which is capable of reaching nearly all mediastinal lymph node stations, including stations 8 and 9, except in cases with pleural adhesions. However, VATS is used only to assess ipsilateral disease. The median sensitivity is 99%, with an NPV of 96% and a 4% false negative rate [40]. All of these surgical techniques are safe, with low morbidity (2%) and mortality (<0.3%) rates. The most common complication is recurrent laryngeal nerve palsy [25,40,41].

In recent years, two new surgical staging techniques have been developed for transcervical lymphadenectomy: VAM lymphadenectomy and transcervical extended mediastinal lymphadenectomy. These techniques permit complete excision of the mediastinal nodes, with a high sensitivity (>95%) and a precision close to 99%, although morbidity rates are higher than with other techniques, ranging from 4% to 6.6%. At present, the use of these techniques is limited to clinical trials [38,42,43].

Invasive mediastinal re-staging after neoadjuvant treatment is complicated because performing a second mediastinoscopy can be difficult due to the presence of treatment-related adhesions and fibrosis; however, when feasible, this procedure offers important advantages in that it allows for the collection of sufficient histological material to accurately restage the patient. Remediastinoscopy for restaging after induction therapy is feasible, with a sensitivity ranging from 61% to 74%, an NPV of 79% to 85%, and a diagnostic accuracy of 88% [44,45]. Restaging with VATS yields a sensitivity and NPV of 83% and 64%, respectively [46]. For both techniques, the specificity is 100%, with minimal morbidity and mortality.

## 4. Role of conventional neoadjuvant treatment: Chemotherapy and chemoradiotherapy

Neoadjuvant therapy plays an important role in reducing tumor size, thus increasing the potential for complete resection while also eliminating micrometastatic disease. Moreover, evaluation of the resected surgical specimen can help to assess treatment response after neoadjuvant therapy, thereby providing valuable prognostic information. To date, however, no consensus has been reached with regard to the optimal induction therapy.

Neoadjuvant chemotherapy (NACT) has shown a survival benefit versus surgery alone in several phase 3 trials, probably due to the high risk of distant metastasis associated with the latter approach. A large meta-analysis (15 studies) compared upfront surgery to platinum-based NACT. The results, published in 2014, showed that the addition of pre-operative chemotherapy improved 5-year overall survival (OS) by 5% (from 40% to 45%) [47]. However, NACT alone does not appear to be sufficient, since the pathological complete response (pCR) rate was low (<10%) and the local and regional recurrence rates (24% and 31%, respectively) were high [48-50].

Given these results, and considering that the optimal induction scheme remains unknown, it was thought that increased local control would improve survival. Studies comparing induction CRT to chemotherapy alone have reported better pCR (60–80%) and mediastinal downstaging rates (53–68%) with higher rates of R0 resections in the CRT arm [51-56]. Both the pCR and mediastinal downstaging rates increased to 75-89% and ≈ndre respectively, in patients who received high-dose neoadjuvant radiotherapy, with low morbidity and mortality rates [57-59]. In recent years, several meta-analyses have compared these neoadjuvant treatment strategies, once again confirming the positive impact of radiotherapy on local control, tumor downstaging, and pCR in the mediastinum, although without finding any significant influence on survival [54,60,61].

## 5. Role of surgery after neoadjuvant treatment

One of the most influential studies to evaluate the role of surgery after neoadjuvant treatment in Stage IIIA-N2 NSCLC was the North American Intergroup trial (INT0139) [7]. However, that trial was unable to demonstrate a clear advantage in OS for induction therapy followed by surgery versus standard definitive CRT; however, as in other phase 3 RCTs [62,63], a subsequent analysis showed a higher 5-year DFS in patients who underwent lobectomy versus pneumonectomy. Multiple retrospective studies and meta-analyses have confirmed these findings [61,64-67].

The standard surgical procedure after neoadjuvant treatment in these patients is complete resection (R0) through lobectomy or pneumonectomy with mediastinal lymphadenectomy. Complete resection is the aim of these multimodal cancer treatments, with 5-year OS rates in patients with R0 close to 40% [68-70].

Compared to lobectomy, pneumonectomy is associated with a greater risk of perioperative morbidity and mortality after induction therapy, which could explain the lack of survival benefit. However, surgical series have shown perioperative mortality rates of 3%-7% after multimodal treatment [51,71-73].

Tumor response to induction therapy is an important prognostic factor. Patients who achieve mediastinal downstaging (ypN0) show better DFS outcomes, with 5-year OS rates > 50%, mainly after lobectomy. However, in patients who respond to induction therapy but have persistent residual mediastinal disease, surgical treatment is feasible provided that complete surgical resection can be achieved, conferring a prognostic improvement [70,74-77].

Therefore, proper selection of surgical candidates within the multimodal treatment approach is crucial. Of course, the patient’s general physical condition and respiratory/cardiovascular function must be taken into account to minimize the risk of morbidity and mortality.

## 6. Role of post-operative radiotherapy (PORT)

The role of PORT in Stage IIIA-N2 NSCLC is controversial. In cases with incompletely resected disease, data support the use of PORT is strong, showing that this approach improves OS [78]. However, in completely resected Stage IIIA-N2 NSCLC, PORT has been a subject of intense debate for more than two decades, ever since a meta-analysis [79] cast doubts on the benefits associated with this approach. Nevertheless, more recent data suggest that not only is PORT not detrimental but also rather it may benefit patients with resected stage IIIA-N2 disease [80-84]. In those studies, the groups were carefully selected, modern radiotherapy techniques and doses were used, the patients received adjuvant chemotherapy and were properly stratified according to the number of nodal metastases. Notwithstanding the findings of those studies, the Lung ART RCT found no benefit for PORT [85]. In that trial, 501 patients with completely resected NSCLC with pathologically-confirmed N2 disease were randomized to receive PORT (54 Gy/27–30 fractions) or no PORT. The 3-year DFS and OS with PORT versus no PORT was 47.1% versus 43.8% and 66.5% versus 68.5%, respectively, but these differences were not statistically significant. Given these contradictory reports, more research is needed to better determine the patient profile most likely to benefit from PORT.

## 7. Concomitant chemoradiotherapy

Curative-intent concomitant platinum-based CRT is the standard treatment in patients with inoperable, locally-advanced NSCLC. Studies have shown that, compared to sequential administration of these two treatments, the concomitant approach improves OS—with an absolute benefit of 5.7% and 4.5% at 3 and 5 years, respectively [5]—and local control, although with increased acute esophageal toxicity [86]. Consolidation treatment with durvalumab (a PD-L1 inhibitor) has been shown to improve both DFS and OS [6]. Consequently, durvalumab was recently added to the standard of care for patients without disease progression after concomitant CRT.

The standard radiotherapy dose ranges from 60 to 66 Gy delivered in conventional fractionation regimens over a 6–7 week period. Dose escalation has been widely evaluated in non-randomized studies, showing a positive impact on local control [87-89]. The RTOG 0617 trial [90] evaluated dose escalation in 464 patients randomized to concomitant treatment with either standard (60 Gy) or high dose radiotherapy (up to 74 Gy). In that trial, oncological results in the lower dose group were not inferior to the high-dose group, which had a higher mortality rate associated with treatment-induced toxicity. A secondary analysis showed that intensity-modulated radiotherapy was associated with a significant reduction in pulmonary toxicity and lower cardiac doses compared to three-dimensional conformal radiotherapy (3D-CRT) [91].

The optimal treatment regimen for chemotherapy administered concurrently with thoracic radiation remains unknown. At present, the standard recommendation is 2–4 cycles of platinum-based chemotherapy delivered concomitantly with radiotherapy. The two most common regimens are cisplatin/etoposide and carboplatin/paclitaxel; although the former regimen has a survival advantage, the latter has a better side effect profile [92-93]. A phase 3 trial found that cisplatin/pemetrexed administered concomitantly with radiotherapy was not superior to standard regimens [94].

Importantly, advanced age does not justify suboptimal treatment. Patients should receive the standard of care provided that their performance status and comorbidities allow for this. In “unfit” patients, a reasonable option could either be sequential treatment or the clinician could consider accelerated radiotherapy versus standard radiotherapy alone [20].

## 8. Immunotherapy and targeted therapies

The efficacy of immunotherapy and targeted therapies for the treatment of Stage IV NSCLC has been well-established [95], leading to a growing number of studies to assess these new therapies in patients locally-advanced disease.

### 8.1. Targeted therapies

Several clinical trials have been performed to determine whether adjuvant epidermal growth factor receptor (EGFR) tyrosine kinase inhibitors (TKI) improve outcomes in early-stage EGFR-mutated lung cancer. However, the phase 3 RADIANT trial [96] compared adjuvant erlotinib to placebo in patients with completely resected, EGFR-positive Stage IB-IIIA NSCLC, found no significant improvement in DFS in the treatment arm versus placebo. However, a *post hoc* analysis of the patient subgroup with EGFR-activating mutations (EGFRm-positive), a small subset of the full study population, found a trend toward better DFS with erlotinib (median DFS, 46 vs. 29 months). Phase II clinical trials have demonstrated better 2-year DFS in patients with EGFRm-positive resected stage locally-advanced NSCLC treated with adjuvant erlotinib versus chemotherapy (81.4% vs. 44.6%), with a better tolerability profile [97,98]. These findings were confirmed in the Phase III ADJUVANT/CTONG1104 trial [99], which compared adjuvant platinum doublet chemotherapy to gefitinib in completely-resected, EGFR-mutated Stage IB-IIIA NSCLC. However, the benefits of treatment appeared to decrease over time, with no clear between-group differences in long-term DFS (28.7 vs. 18 months, respectively), suggesting that treatment with gefitinib delayed but did not necessarily prevent recurrence. New data from that trial confirm that the improved DFS did not translate to better OS; at a median follow-up of 76.9 months, there were no significant differences in median OS between the two arms (75.5 vs. 79.2 months, HR 0.92) [100]. Therefore, it is not clear whether adjuvant treatment with EGFR TKIs can alter the natural history of the disease to improve cure rates, or whether they simply delay recurrence. The results of the Phase III ADAURA trial [101] comparing 3 years of adjuvant osimertinib to placebo in patients with completely-resected, EGFR-mutated Stage IB-IIIA NSCLC were recently published. The 2-year DFS was statistically significant, with a clinically meaningful improvement in DFS in the osimertinib group (90% vs. 40%, respectively). Despite these promising findings, OS data are needed before osimertinib can be considered standard of care in these patients.

EGFR TKIs have also been evaluated as induction therapy in patients with a molecularly-selected population of patients with potentially-resectable NSCLC, demonstrating good tolerability and safety but uneven results in terms of objective response rate (ORR) and survival [102,103]. A small Phase II trial evaluated the efficacy of neoadjuvant erlotinib in patients with EGFR-mutated Stage IIIA NSCLC. After surgery, the patients who received erlotinib had a marginally better clinical ORR (67% vs. 19%), pathological response rate (67% vs. 38%), and OS (51.0 vs. 20.9 months) compared with those who received chemotherapy [104]. Another multicenter study, EMERGING-CTONG 1103, reported a significant improvement in DFS with erlotinib versus gemcitabine-cisplatin chemotherapy (21.5 vs. 11.4 months; HR 0.39) in the same group of patients [105]. The ASCENT trial [106] compared afatinib, a second-generation EGFR TKI, to standard CRT in the neoadjuvant setting in patients with Stage III NSCLC. Patients who received neoadjuvant afatinib had high overall response (69%) and major pathologic response (MPR) rates to surgery. That trial is still underway, as is the neo ADAURA trial (NCT04351555).

Erlotinib combined with radiotherapy may be more effective than CRT alone in Stage III lung cancer, thus obviating the need for chemotherapy in EGFRm-positive patients [107], but Phase III trials are needed to confirm this hypothesis. To date, none of the targeted therapies in combination with CRT in locally-advanced NSCLC have shown a survival benefit.

The anaplastic lymphoma kinase (ALK) fusion oncogene is another predictive biomarker identified in a small subset of patients with NSCLC. For this reason, recruitment in neoadjuvant and adjuvant studies is difficult. In the neoadjuvant setting, one study is currently evaluating the role of ALK TKIs. One multicenter Phase II trial (NCT04302025) is evaluating the efficacy of 8 weeks of targeted therapy (alectinib, entrectinib, vemurafenib, or cobimetinib) in patients with Stage IB-IIIB NSCLC with various different molecular alterations (ALK-rearranged; ROS1-rearranged, NTRK-rearranged, and BRAF mutated). In terms of adjuvant therapy, the ongoing Phase III ALINA trial (NCT 03456076) is investigating the efficacy and safety of adjuvant alectinib versus chemotherapy in completely-resected, ALK-rearranged Stage IB-IIIA NSCLC.

### 8.2. Immunotherapy

Immunotherapy with immune checkpoint inhibitors (ICI) has revolutionized the therapeutic approach to NSCLC, primarily through the use of human IgG1 monoclonal antibodies that block programmed death receptor 1 (PD-1) and its ligand (PD-L1). These checkpoint inhibitors are associated with higher response rates, improved OS, and better tolerability when compared to conventional cytotoxic chemotherapy. Not surprisingly, this has generated increased interest in expanding the use of ICIs in earlier, resectable stages of NSCLC to prevent recurrences and improve cure rates.

#### 8.2.1. Neoadjuvant immunotherapy

Preclinical data suggest that neoadjuvant immunotherapy is more effective than adjuvant immunotherapy, perhaps due to the more immunogenic tumor microenvironment versus that of post-surgical micrometastases [108]. From a biological perspective, pre-operative PD-1/PD-L1 blockade, when the tumor and locoregional lymph nodes are still present and can interact dynamically with immune cells, may be a more rational approach [109,110]. Multiple ICIs have been evaluated in combination with neoadjuvant therapy, and the positive results of these studies have demonstrated the feasibility and safety of this neoadjuvant approach in NSCLC (Tables 1 and 2).

**Table 1:** Ongoing Phase II Clinical Trials of Neoadjuvant Immunotherapy and Radiotherapy in NSCLC

	Eligible patients	Intervention	Estimated enrolment	Primary endpoint
NCT03237377	Resectable IIIA	Arm A: Durvalumab+Radiation (45 Gy/25 fx)→Surgery Arm B: Durvalumab+Tremelimumab+Radiation (45 Gy/25 fx)→Surgery	*n*=32	Toxicities and Feasibility
CHIO3 NCT04062708	Resectable IIIA/B	Platinum doublet×4 cycles+Durvalumab→Surgery±PORT (54Gy)+Durvalumab×13 cycles.	*n*=55	Nodal clearance
NCT03871153	Resectable III N2	Durvalumab+Paclitaxel+Carboplatin+RT (45–61.2 Gy)+Durvalumab→Surgery→Durvalumab	*n*=25	pCR
NCT02572843	Resectable IIIA N2	Cisplatin/Docetaxel×3 cycles→IT MEDI4736 (anti-PD-L1)→Surgery (±PORT) r IT MEDI4736 (anti-PD-L1)	*n*=68	EFS

PORT: Post-operative radiotherapy, pCR: Pathological complete response, EFS: Event-free survival, IT: Immunotherapy.

**Table 2:** Ongoing Phase II Trials of Neoadjuvant Immunotherapy with or without Chemotherapy in NSCLC

	Disease stage	N/Resected	Intervention	Primary objective	MPR (%)/pCR (%)	Surgery (%)
NADIM NCT03081689 [122]	IIIA (N2 or T4)	46/41	Nivolumab+Paclitaxel+Carboplatin→Surgery→Nivolumab 1 year	PFS at 24 months	83/59	89
SKCCC-JHU NCT02259621 [111]	IB - IIIA	22/21	Nivolumab×2 cycles→Surgery.	Safety Feasibility	45/15	95
NEOSTAR NCT03158129 [108]	I - IIIA (N2 only)	88/N: 23, N-I: 21	Arm A: Nivolumab→Surgery. Arm B: Nivolumab, Ipilimumab→Surgery.	MPR	N: 17/9 N-I: 33/29	N: 96 N-I: 81
LCMC3 NCT02927301[110]	IB-IIIB (T3N2)	90/77	Atezolizumab×2 cycles→Surgery→Atezolizumab 1 year	MRP	19/5	89
Columbia University NCT02716038[123]	IB - IIIA	30/11	Atezolizumab+Carboplatin+Nab-paclitaxel→Surgery.	MPR	57/33	87
SAKK 16/14 NCT02572843 [124]	IIIA (T1-3 N2 M0)	68/55	Cisplatin+Docetaxel×3 cycles→Durvalumab×2 cycles→Surgery→Durvalumab 1 year	EFS	60/18.2	81

MPR: Major pathologic response, pCR: Pathological complete response, PFS: Progression-free survival, N: Nivolumab, I: Ipilimumab, EFS: Event-free survival

The efficacy and safety of neoadjuvant nivolumab have been evaluated in patients with surgically resectable NSCLC, with few side effects and high MPR rates [111]. In that study, the mutational burden of the tumor was predictive of the pathological response to PD-1 blockade. The first clinical study to explore the safety and antitumor activity of neoadjuvant chemo-immunotherapy (paclitaxel – carboplatin plus nivolumab) in resectable stage IIIA NSCLC was the NADIM trial [112]. At 24 months, the DFS (primary endpoint) was 77.1%, with an OS rate of 90%. All patients who underwent surgery showed an MPR, with 63% having a pCR A new Phase II RCT (NADIM-2) is currently in progress to compare the same neoadjuvant chemo-immunotherapy regimen followed by a shorter (6 months) adjuvant nivolumab monotherapy versus standard chemotherapy.

Neoadjuvant ICI appears to be a promising therapeutic option for patients with resectable stage IIIA-N2 disease, but this treatment requires confirmation in a RCT. Several Phase III trials of neoadjuvant immunotherapy are currently underway (Table 3).

**Table 3:** Ongoing Randomized Phase III Trials of Neoadjuvant Immunotherapy With or Without Chemotherapy in NSCLC

	Eligible patients	Intervention	Estimated enrolment	The primary endpoint
ChekMate 816 NCT02998528	IB-IIIA	Arm A: Platinum doublet × 3 cycles → Surgery → CT ± RT Arm B: Nivolumab + Platinum doublet × 3 cycles → Surgery → CT ± RT	*n*=350	DFS pCR
IMpower030 NCT03456063	II, IIIA, select IIIB	Arm A: Atezolizumab + Platinum doublet × 4 cycles → Surgery → Atezolizumab Arm B: Placebo + Platinum doublet × 4 cycles → Surgery → Placebo	*n*=302	MPR DFS
KEYNOTE 671 NCT03425643	IIB-IIIA	Arm A: Pembrolizumab + Platinum doublet × 4 cycles → Surgery → Pembrolizumab. Arm B: Placebo + Platinum doublet × 4 cycles → Surgery → Placebo	*n*=786	DFS OS
ChecMate 77T NCT04025879	II-IIIB	Arm A: CT + Nivolumab → Surgery + Nivolumab Arm B: CT + Placebo → Surgery + Placebo	*n*=452	DFS
AEGEAN NCT03800134	IIIA-IIIB	Arm: CT + Durvalumab → Surgery Arm B: CT + Placebo → Surgery	*n*=300	MPR

CT: Chemotherapy, RT: Radiotherapy, DFS: Disease-free survival, pCR: Pathological complete response, MPR: Major pathological response, OS: Overall survival

#### 8.2.2. Adjuvant immunotherapy

In recent decades, several different adjuvant treatments developed to improve prognosis in patients with resected NSCLC have been evaluated. Adjuvant cisplatin-based chemotherapy is now standard of care for patients with Stage II–IIIA disease [113,114], but survival rates remain poor. Novel therapeutic strategies, such as vaccines, have also been studied. The MAGRIT trial was the first study to evaluate adjuvant immunotherapy in patients with resected NSCLC. In that trial, 2272 patients with completely resected Stage IB-II or IIIA NSCLC (with and without adjuvant chemotherapy) with positive MAGE-A3 expression were randomly assigned (2:1) to receive either MAGE-A3 immunotherapy or placebo. Unfortunately, MAGE-A3 did not yield any significant improvement in DFS (60.5 vs. 56.9 months, HR 1.02) [115]. Several clinical trials are currently evaluating the role of adjuvant immunotherapy, but no results have been published to date (Table 4).

**Table 4:** Ongoing Randomized Phase III Trials of Adjuvant Immunotherapy in Early-Stage and Locally-Advanced NSCLC

	Eligible patients	Intervention	Estimated enrolment	Primary endpoint
ANVIL NCT02595944	IB-IIIA	Arm A: Surgery→CT→Nivolumab 1 year Arm B: Surgery→CT→Observation.	*n*=903	DFS OS
IMpower010 NCT02486718	II, IIIA, select IIIB	Arm A: Surgery→Platinum doublet×4 cycles→Atezolizumab×16 cycles. Arm B: Surgery→Platinum doublet×4 cycles→Observation.	*n*=1280	DFS OS
KEYNOTE 091-PEARLS NCT02504372	IB/II-IIIA	Arm A: Surgery→±CT→Pembrolizumab 1 year Arm B: Surgeryi±CT→Placebo 1 year	*n*=1080	DFS
BR-31 NCT02273375	IB-IIIA	Arm A: Surgery→±CT→Durvalumab 1 year Arm B: Surgery→±CT→Placebo 1 year	*n*=1360	DFS
CANOPY-A NCT03447769	II -IIIA and IIIB (T>5cm N2)	Arm A: Surgery→±CT→Canakinumab×18 cycles. Arm B: Surgery→±CT→Placebo 18 cycles.	*n*=1500	DFS

CT: Chemotherapy, RT: Radiotherapy, DFS: Disease-free survival, OS: Overall survival

#### 8.2.3. Immunotherapy in inoperable locally-advanced NSCLC

The results of the Phase III PACIFIC trial, published in 2018 [6], demonstrated a 2-year OS of 66.3% in Stage III NSCLC patients treated with CRT, followed by durvalumab versus only 55.6% in the placebo group (*P* = 0.0025), with a statistically significant improvement in PFS. Crucially, the results of that trial changed the standard of care in this patient population. Treatment was well-tolerated, although there was a trend towards more pneumonitis in the durvalumab group (33.9% vs. 24.8%, respectively); however, the rate of Grade 3 or higher pneumonitis was similar. A follow-up study reported 3-year survival data demonstrating the long-term clinical benefits of consolidation therapy with durvalumab, with a 3-year OS of 55% in the durvalumab group versus 44% in the placebo group, and 4-year OS rates of 49.6% versus 36.3%, respectively [116,117]. Treatment with durvalumab also prolonged time to distant metastasis and decreased the incidence of new brain metastases versus placebo (6.3% vs. 11.8%) [117]. In the PACIFIC trial, PD-L1 testing was not mandatory and thus PD-L1 status was unknown in 37% of patients. An exploratory analysis showed a survival benefit (OS) in patients who presented PD-L1 expression ≥1%; by contrast, treatment with durvalumab had a detrimental effect in patients without PD-L1 expression. The efficacy of durvalumab in real-world settings is currently being evaluated in the PACIFIC-R trial (NCT03798535) [118]. Other unresolved questions are being evaluated in the Phase III PACIFIC5 trial, which is comparing a flat dose of durvalumab to placebo after concurrent or sequential CRT, and in the Phase II PACIFIC6 trial (NCT03693300), which is evaluating durvalumab after sequential treatment.

Other immunotherapy agents, such as pembrolizumab, have shown survival outcomes similar to those reported in the PACIFIC study. For example, a small, single-arm Phase II trial (LUN 14-179) [119] evaluated consolidation pembrolizumab after CRT (up to 12 months), finding that the time to metastatic disease or death was 30.7 months, which was significantly longer (*P* < 0.0001) than a historical controls. The median PFS was 18.7 months and rates of Grades 3–5 pneumonitis were slightly higher compared to durvalumab [119].

Several studies are currently underway to investigate the synergistic effect of radiotherapy combined with immunotherapy, with promising initial results. In addition, several trials are underway to evaluate the efficacy and (especially) the safety of immunotherapy administered concurrently with standard CRT in locally-advanced Stage IIIA/B NSCLC [120,121]. (Tables 5 and 6)

**Table 5:** Ongoing Randomized Phase III Trials of Immunotherapy and Chemoradiotherapy in Locally-Advanced NSCLC

	Eligible patients	Intervention	Estimated enrolment	Primary endpoint
CHECKMATE 73L NCT04026412	III	Arm A: Nivolumab + CRT → Nivolumab + Ipilimumab. Arm B: Nivolumab + CRT → Nivolumab. Arm C: Nivolumab + CRT → Durvalumab.	*n*=1400	DFS OS
PACIFIC 2 NCT03519971.	III	Arm A: Durvalumab + CRT → Durvalumab. Arm B: Placebo + CRT → Placebo.	*n*=328	DFS ORR
KEYNOTE 799 NCT03631784 * phase II trial	III	Cohort A: Pembrolizumab + Carboplatin + Paclitaxel × 1 cycles → Pembrolizumab × 2 cycles + weekly carbo/paclitaxel + RT → Pembrolizumab × 14 cycles. Cohort B: Pembrolizumab + Cisplatin + Pemetrexed × 3 cycles + RT → Pembrolizumab × 14 cycles.	*n*=210	Grade > 3 Pneumonitis ORR
EA5181 NCT04092283	III	Arm A: durvalumab + CRT → Durvalumab. Arm B: CRT → Durvalumab.	*n*=660	OS

CRT: Chemoradiotherapy, RT: Radiotherapy, pCR: Pathological complete response, MPR: Major pathological response, OS: Overall survival, DFS: Disease-free survival, ORR: Objective response rate

**Table 6:** Results of Studies Combining Chemoradiotherapy with Immunotherapy in Stage III NSCLC

	*n*	Intervention	DFS	OS (%)	Toxicity>Grade 3 (%)
PACIFIC [6,117]	714	CRT (54–66 Gy)→Durvalumab.	Median, 18.8 months	1 year: 83.1 2 year: 66.3	30.5
LUN 14-179 [119]	92	CRT (59.4–66 Gy)→Pembrolizumab.	Median, 15.4 months	1 year: 80.5 2 year: 68.7	6.5
ETOP NICOLAS [121,125]	80	CRT (66 Gy)+Nivolumab→Nivolumab 1 year	1 year: 54%	1 year: 79	10.9
DETERRED [120]	40	Part 1: CRT (60–66 Gy)→CT+Atezolizumab×2 cycles→Atezolizumab 1 year Part 2: CRT (60–66 Gy)+Atezolizumab→CT+Atezolizumab×2 cycles→Atezolizumab 1 year	1 year: 57%	1 year: 79	27.5

DFS: Disease-free survival, OS: Overall survival, CRT: Chemoradiotherapy, CT: Chemotherapy

## 9. Conclusions

Patients with Stage IIIA-N2 NSCLC are a highly heterogeneous group, but all of them require combined treatment modalities to ensure locoregional and systemic disease control. Local treatments include radiotherapy and surgery while systemic therapies include chemotherapy, immunotherapy, and targeted therapies. To optimize disease control in these patients, accurate staging is essential.

PET-CT imaging provides both morphological and functional data, thus offering a more efficient method to detect mediastinal involvement and to achieve early diagnosis of local and distant recurrence. Minimally-invasive endoscopic techniques allow for cytological/histological confirmation of mediastinal involvement, thus avoiding unnecessary initial thoracotomies and reducing the time to diagnosis. All these advances enable an increasingly precise clinical subclassification of Stage IIIA N2 patients, although the optimal treatment for each subgroup remains controversial.

Currently available data on the various treatment combinations and sequences, both local and systemic, provide little clarity with regards to the most appropriate strategy to maximize survival outcomes by improving disease control while minimizing treatment-related toxicity. In most patients with resectable disease, multimodal treatment plays an important role. Although the optimal induction therapy regimen is still under debate, surgery — especially lobectomy — has been shown to improve both local control and OS, mainly in patients who present a good tumor response to neoadjuvant treatment with mediastinal downstaging.

Newer radiotherapy techniques improve tumor coverage and provide highly conformal radiation doses to the treatment volume while minimizing doses to the organs at risk, thereby allowing for higher radiation doses with less toxicity and a lower impact on QoL. Despite technological advances in radiotherapy combined with third-generation chemotherapy agents, survival rates in patients with inoperable or unresectable stage IIIA NSCLC treated with concomitant CRT have not improved. In fact, the real revolution in the treatment of this disease is the recent emergence of targeted therapies and immunotherapy, both of which are ready for inclusion in this multimodal treatment approach. At present, the use of targeted agents in Stage III NSCLC is limited to clinical trials. Studies that have assessed the addition of immunotherapy to induction therapy have shown highly promising results. Even so, more studies are needed to determine the optimal treatment approach for the various patient subgroups with Stage IIIA NSCLC.

### Conflict of interest

The authors indicate no potential conflicts of interest.
